# Minding the consumer mindsets in addressing gambling consumption harms

**DOI:** 10.3389/fpsyg.2022.905785

**Published:** 2022-08-22

**Authors:** En Li, Hannah Thorne, Matthew Browne, Matthew Rockloff

**Affiliations:** ^1^School of Business and Law, Central Queensland University, Rockhampton, QLD, Australia; ^2^School of Health, Medical and Applied Sciences, Central Queensland University, Adelaide, SA, Australia; ^3^School of Health, Medical and Applied Sciences, Central Queensland University, Bundaberg, QLD, Australia

**Keywords:** consumer mindset, fresh start mindset, busy mindset, short-term mating mindset, gambling, gambling consumption harms

## Introduction

Gambling is a widely accessible form of consumption across numerous jurisdictions around the world (Orford, 2020), with its global market size reaching hundreds of billions of dollars annually (Research and Markets, 2021). Despite this mass popularity of gambling consumption internationally, it has been well-recognized that such consumption can have harmful consequences on various aspects of individual consumers' life, such as their finance, health, relationship, work, or study (e.g., Li et al., 2017). The purpose of this paper is to discuss some of the latest findings in consumer psychology/behavior on consumer mindsets, and to provide critical analysis on how consumer mindsets could be potentially leveraged to address harms from gambling consumption. Based on the consumer psychology literature (Rucker and Galinsky, 2016), a consumer mindset can be defined as a psychological orientation that affects consumer information processing, consumer evaluations, and consumer responses. Three specific consumer mindsets will be discussed below as exemplars which can have direct relevance to the pathways to reduce or prevent gambling consumption harms, including the fresh start mindset (Price et al., 2018), the busy mindset (Kim et al., 2019), and the short-term mating mindset (He and Cunha, 2020).

## Three consumer mindsets

### Fresh start mindset

The fresh start mindset is “defined as a belief that people can make a new start, get a new beginning, and chart a new course in life, regardless of past or present circumstances” (Price et al., 2018, p. 22). Consumers with a stronger fresh start mindset, were found to exert larger amounts of effort into a variety of transformative activities, including those activities related to transforming their budget, health, relationship, and consumption practices (Price et al., 2018). Furthermore, Price et al. (2018) suggested that the fresh start mindset could be activated through a great deal of natural stimuli, such as marketing promotions and news articles. In an experimental study (Price et al., 2018), they randomly assigned their participants to one of the following three conditions: a first condition where the participants viewed a news article on fresh starts being possible for most people; a second condition where the participants viewed a news article on fresh starts being not possible for most people; and a third control condition where the participants had no exposure to the information on fresh starts. It was found that the participants' fresh start mindset measurement was indeed affected by this mindset manipulation procedure: the participants in the first condition reported significantly higher level of fresh start mindset measurement compared to those in the third control condition, who in turn, reported significantly higher fresh start mindset measurement level than those in the second condition (Price et al., 2018). In addition, the mindset manipulation procedure had further impacts on multiple downstream consumer behavior relevant variables: the participants evaluated a sunglasses advertisement which featured the headline “New look. New you.” (Price et al., 2018, p. 36), and significant indirect effects of the mindset manipulation *via* the fresh start mindset measurement were found on outcomes including the participants' attitude to the advertisement, their likelihood to buy the sunglasses in the advertisement, and the log-transformed value of the amount they were willing to pay for the sunglasses (Price et al., 2018).

Given the evidence discussed above, the fresh start mindset could be utilized in successful ways to counter gambling consumption harms. Specifically, when designing campaigns to promote those products or services which aim to reduce or prevent gambling consumption harms, marketers should consider when or how to potentially increase the likelihood of the fresh start mindset activation among their target customers. Dai et al. (2015) discovered that a variety of temporal landmarks can be highlighted or signified as the new beginning (e.g., the first day of a season, the first day of an organization's break period, and the first day after moving to a new place as the first move in a long time), and that emphasizing such temporal landmarks can increase individuals' intention to begin goal pursuits. Hence, promotions of gambling harm reduction/prevention products or services could benefit from incorporating and highlighting certain temporal landmarks. For instance, marketers of these products or services could encourage consumers who experience or are at risks of gambling consumption harms to adopt or trial their products or services at the start of a new year/season/month/week, or on those consumers' birthdays, anniversaries, or other milestone days. Importantly, the marketers should emphasize in their promotional messaging that these suggested temporal landmarks signify the new beginning and the products or services being promoted can benefit or lead to a “new you”. The marketers can also feature in their promotional campaigns success stories, past customer testimonials, or corresponding statistics, to showcase the real possibility of getting a fresh start and steering away from gambling consumption harms.

### Busy mindset

The concept of busy mindset refers to the subjective perception of oneself as busy (Kim et al., 2019). Across multiple studies, Kim et al. (2019) found support that a busy mindset could be activated or induced through several ways, including using simple cues such as an advertising tagline that had the word “busy” in it. Kim et al. (2019) also discovered that the activation of a busy mindset could boost individuals' self-control abilities in various contexts, such as reducing their likelihood of getting indulgent food, and increasing the percentage of their income they would save. In addition, Kim et al. (2019) demonstrated the evidence that the boosting effect of busy mindset activation on self-control was driven by self-importance, such that individuals with an induced busy mindset reported increased feeling of self-importance, which subsequently increased their saving percentage. Thus, decisions made when in a busy mindset may be guided by a desire to act in a way that is congruent with heightened self-importance, prioritizing self-control that may lead to the preference of long-term gain over instant gratification of hedonic impulse (Kim et al., 2019).

Self-control issues are considered to be “at the heart of problem gambling” (Bergen et al., 2012, p. 637). Further, a lower level of self-control can be associated with a higher level of problem gambling severity (Bergen et al., 2012). Hence, the boosting effect of busy mindset activation on self-control could be leveraged to potentially address gambling consumption harms. For example, a diverse range of responsible gambling tools have been existing (e.g., self-exclusion, spending and time limits, Wood et al., 2014), which ideally could offer consumer protection against potential gambling problems. However, evidence has revealed low uptakes of responsible gambling tools, such as those resources on self-exclusion or spending/deposit limits (Gainsbury et al., 2018). Therefore, it might facilitate the adoption of responsible gambling tools, if a busy mindset could be activated or induced in the immediate contexts where such adoption decisions are made by consumers (e.g., through presenting busyness related words or signs in venues or on websites where those responsible gambling tools are offered to consumers). In addition, McAuliffe et al. (2021) reviewed a number of structural feature tools, user-directed tools, and regulatory initiatives, and found inconsistency with regards to their impacts. For instance, they identified 20 studies which suggested favorable responsible gambling impact from pop-up messages, but also seven studies suggesting no responsible gambling impact from pop-up messages and 14 studies suggesting mixed impact from pop-up messages (McAuliffe et al., 2021). Hence, it would be worthwhile to test if busy mindset activation could boost the effectiveness of those specific tools or initiatives, particularly those ones that have been found to offer mixed or even no impact.

### Short-term mating mindset

He and Cunha (2020) argued that individuals under a short-term mating state would make adaptations to collect information on or attract potential mating partners (for later scrutinizing if needed), and would prioritize finding the potential partners' strengths rather than fending off their weaknesses. Further, He and Cunha (2020) predicted that the short-term mating state would cause individuals to act instead of accepting their default or existing option. It has been discovered that a short-term mating mindset could be activated among study participants who imagined a scenario about a blind date and answered questions on their dating related preferences, and that those under an activated short-term mating mindset could exhibit increased tendency toward choosing non-default over default options (He and Cunha, 2020). For instance, when participants in an experiment were given a choice between two investment options (i.e., between less risky bonds and more risky stocks), the participants with (vs. those without) an activated short-term mating mindset, turned out to display a lower likelihood of choosing stocks when stocks were described as the default or existing investment situation, and display a higher likelihood of choosing stocks when stocks were not the default (He and Cunha, 2020).

A preference for the default situation or the status quo seems to be present in consumers who experience negative impacts associated with gambling, with only a small percentage or a minority of pathological gamblers seeking help or treatment (Braun et al., 2014). This preference for the status quo represents a lost opportunity to reduce harm from gambling. It has been found that a significant risk factor for pathological gambling can be younger age (Johansson et al., 2009), a demographic that lends itself to the incidental activation of short-term mating mindset due to the cues often present within the environment in which young people operate. Mobile dating applications (e.g., Tinder) have been quite popular among young adult consumers (Sawyer et al., 2018). In browsing these applications, young consumers are likely being primed with a short-term mating mindset. A within-dating-application advertisement that portrays living with gambling problems as the default or status quo and engaging with treatment services as the action to change the status quo may increase the likelihood of young consumers accessing treatment, when paired with a short-term mating mindset. This may be especially the case if the action step is easily accessible, such as real-time online chat counseling (Rodda et al., 2017).

## Discussion

Research on gambling consumption harms has been gaining momentum in recent years (e.g., Li et al., 2017; Jeffrey et al., 2019; Booth et al., 2021), but this line of research has largely overlooked the potentially beneficial roles of consumer mindsets in addressing gambling consumption harms. All three consumer mindsets discussed here, as well as the likely ways by which they can be utilized to reduce or prevent gambling consumption harms, are warranted to be evaluated empirically. In addition, given different gambling forms/activities could be associated with different risk functions or levels of harm (Markham et al., 2016; Livingstone and Rintoul, 2020), the potential protective effects of consumer mindsets should be compared among consumers of different gambling activities. Moreover, since a substantial proportion of the heaviest gambling spenders “suffer from addictions, mental and physical health issues, and are otherwise vulnerable” (Sulkunen, 2022, p. 63), and a public health approach would pay particular attention to those most at risk of harm (van Schalkwyk et al., 2021), it would be valuable to examine how consumer mindsets could be applied to assist those consumers who spend the most on gambling or are most at risk. According to Sulkunen et al. (2019), “the term ‘harmful gambling' encompasses all degrees of severity and frequency, including ‘problem' and ‘pathological' gambling” (p. 37), and they argued that a public interest approach should take into consideration the full range of gambling's problematic consequences, rather than only those consequences experienced by the gamblers themselves (Sulkunen et al., 2019). Hence, further investigations could also be carried out to test the usefulness of inducing the consumer mindsets in question among gamblers' affected others (i.e., “any person with a significant relationship to a gambler who is affected by the gambler's behavior”, Li et al., 2017, p. 224). Outcomes of these evaluations and investigations will benefit the theoretical advancements of consumer psychology and clinical psychology, as well as the practice of regulators, clinicians, social marketers, and other stakeholders in addressing gambling consumption harms.

## Author contributions

EL: conceptualization. EL and HT: drafting. EL, MB, and MR: critical revisions. All authors contributed to the article and approved the submitted version.

## Funding

HT was supported by the funding from CQUniversity's Enrich Research Employment Initiative when working on this manuscript.

## Conflict of interest

The authors declare that the research was conducted in the absence of any commercial or financial relationships that could be construed as a potential conflict of interest.

## Publisher's note

All claims expressed in this article are solely those of the authors and do not necessarily represent those of their affiliated organizations, or those of the publisher, the editors and the reviewers. Any product that may be evaluated in this article, or claim that may be made by its manufacturer, is not guaranteed or endorsed by the publisher.

## References

[B1] Bergen A. E. Newby-Clark I. R. Brown A. (2012). Low trait self-control in problem gamblers: evidence from self-report and behavioral measures. J. Gamb. Stud. 28, 637–648. 10.1007/s10899-011-9274-9 21989573 PMC3986897

[B2] Booth L. Anderson A. S. White V. Pierce H. Moodie R. Pettigrew S. (2021). Public perceptions of harm for nine popular gambling products. J. Gamb. Stud. 37, 1113–1126. 10.1007/s10899-021-10014-5 33635504

[B3] Braun B. Ludwig M. Sleczka P. Bühringer G. Kraus L. (2014). Gamblers seeking treatment: who does and who doesn't?. J. Behav. Addict. 3, 189–198. 10.1556/JBA.3.2014.3.7 25317343 PMC4189314

[B4] Dai H. Milkman K. L. Riis J. (2015). Put your imperfections behind you: Temporal landmarks spur goal initiation when they signal new beginnings. Psychol. Sci. 26, 1927–1936. 10.1177/0956797615605818 26546079 PMC4839284

[B5] Gainsbury S. M. Abarbanel B. L. L. Philander K. S. Butler J. V. (2018). Strategies to customize responsible gambling messages: a review and focus group study. BMC Public Health 18, 1381. 10.1186/s12889-018-6281-0 30558568 PMC6297977

[B6] He Y. Cunha M. Jr. (2020). Love leads to action: Short-term mating mindset mitigates the status-quo bias by enhancing promotion focus. J. Consum. Psychol. 30, 631–651. 10.1002/jcpy.1174

[B7] Jeffrey L. Browne M. Rawat V. Langham E. Li E. Rockloff M. (2019). Til debt do us part: comparing gambling harms between gamblers and their spouses. J. Gamb. Stud. 35, 1015–1034. 10.1007/s10899-019-09826-3 30701378

[B8] Johansson A. Grant J. E. Kim S. W. Odlaug B. L. Götestam K. G. (2009). Risk factors for problematic gambling: a critical literature review. J. Gamb. Stud. 25, 67–92. 10.1007/s10899-008-9088-6 18392670

[B9] Kim J. C. Wadhwa M. Chattopadhyay A. (2019). When busy is less indulging: impact of busy mindset on self-control behaviors. J. Consum. Res. 45, 933–952. 10.1093/jcr/ucy069

[B10] Li E. Browne M. Rawat V. Langham E. Rockloff M. (2017). Breaking bad: comparing gambling harms among gamblers and affected others. J. Gamb. Stud. 33, 223–248. 10.1007/s10899-016-9632-8 27443306

[B11] Livingstone C. Rintoul A. (2020). Moving on from responsible gambling: a new discourse is needed to prevent and minimise harm from gambling. Public Health 184, 107–112. 10.1016/j.puhe.2020.03.018 32434694

[B12] Markham F. Young M. Doran B. (2016). The relationship between player losses and gambling-related harm: Evidence from nationally representative cross-sectional surveys in four countries. Addiction 111, 320–330. 10.1111/add.13178 26567515

[B13] McAuliffe W. H. B. Edson T. C. Louderback E. R. LaRaja A. LaPlante D. A. (2021). Responsible product design to mitigate excessive gambling: A scoping review and z-curve analysis of replicability. PLoS ONE 16, e0249926. 10.1371/journal.pone.0249926 33878126 PMC8057587

[B14] Orford J. (2020). The Gambling Establishment: Challenging the Power of the Modern Gambling Industry and Its Allies. Abingdon: Routledge.

[B15] Price L. L. Coulter R. A. Strizhakova Y. Schultz A. E. (2018). The fresh start mindset: Transforming consumers' lives. J. Consum. Res. 45, 21–48. 10.1093/jcr/ucx115

[B16] Research Markets (2021). Global Gambling Market Report 2021: Market to Grow From $465.76 Billion in 2020 to $516.03 Billion in 2021 - Forecast to 2030. PR Newswire. Available online at: https://www.prnewswire.com/news-releases/global-gambling-market-report-2021-market-to-grow-from-465-76-billion-in-2020-to-516-03-billion-in-2021—forecast-to-2030–301224701.html (accessed June 30, 2022).

[B17] Rodda S. N. Lubman D. I. Jackson A. C. Dowling N. A. (2017). Improved outcomes following a single session web-based intervention for problem gambling. J. Gamb. Stud. 33, 283–299. 10.1007/s10899-016-9638-2 27562272

[B18] Rucker D. D. Galinsky A. D. (2016). Growing beyond growth: why multiple mindsets matter for consumer behavior. J. Consum. Psychol. 26, 161–164. 10.1016/j.jcps.2015.06.009

[B19] Sawyer A. N. Smith E. R. Benotsch E. G. (2018). Dating application use and sexual risk behavior among young adults. Sex. Res. Soc. Policy 15, 183–191. 10.1007/s13178-017-0297-630728064

[B20] Sulkunen P. (2022). “Where does the gambling surplus come from? Outline of a theory of an asymmetric market,” in The Global Gambling Industry: Structures, Tactics, and Networks of Impact, eds J. Nikkinen, V. Marionneau, and M. Egerer (Wiesbaden: Springer Gabler), 55–67.

[B21] Sulkunen P. Babor T. F. Cisneros Örnberg J. Egerer M. Hellman M. Livingstone C.. (2019). Setting Limits: Gambling, Science and Public Policy. Oxford: Oxford University Press. 10.1111/add.15241 33084199

[B22] van Schalkwyk M. C. I. Petticrew M. Cassidy R. Adams P. McKee M. Reynolds J.. (2021). A public health approach to gambling regulation: countering powerful influences. Lancet Public Health 6, e614–e619. 10.1016/S2468-2667(21)00098-0 34166631

[B23] Wood R. T. A. Shorter G. W. Griffiths M. D. (2014). Rating the suitability of responsible gambling features for specific game types: a resource for optimizing responsible gambling strategy. Int. J. Ment. Health Addict. 12, 94–112. 10.1007/s11469-013-9473-y

